# A Blinded Comparative Study of Four Commercially Available LEDs and a Laser Light Curing Device

**DOI:** 10.1055/s-0042-1757908

**Published:** 2022-12-27

**Authors:** John C. Comisi, Cristiane Maucoski, Jonathan P. Beller, Kyle S. Dennis, Richard B. Price

**Affiliations:** 1 Department of Oral Rehabilitation, James B. Edwards College of Dental Medicine, Medical University of South Carolina, Charleston, South Carolina, United States; 2 Department of Dental Clinical Sciences, Faculty of Dentistry, Dalhousie University, Halifax, Nova Scotia, Canada

**Keywords:** resin-based composites, dental curing lights, LED curing lights, laser curing light

## Abstract

**Objective** This study determined the effectiveness of five light-curing units (LCUs; four light-emitting diode [LED] and one laser) used on different settings to photo-activate four conventional resin-based composites (RBCs).

**Materials and Methods** A total of 108 RBC specimens were photo-activated in a white Delrin mold representing a mesial-occlusal-distal (MOD) class II restoration in a molar tooth. The proximal boxes were 5 mm deep, and the mesial-distal length was 12 mm. Immediately after photo-curing, the RBC specimens were immersed in a solvent to remove the uncured materials, after which they were photographed and deidentified. A Research Electronic Data Capture survey was created using these images and sent to respondents who blindly assessed the ability of the various LCUs to photo-cure the MOD restorations.

**Results** There were significant differences in how the five curing lights had cured RBCs. One-way analysis of variance (ANOVA), pairwise *t* -test, Welch's one-way ANOVA, and Kruskal–Wallis rank test in the blinded survey data showed significant differences between the LED curing lights used for two 10-second cures and the laser curing light used for 1 second, and LED lights at lower settings.

**Conclusion** There was a significant difference in how the curing lights could photo-cure the RBCs used in this study. The laser curing light used for 1 second produced the worst results in all four RBCs.

## Introduction

Light-curing units (LCUs) are used extensively in orthodontics, prosthodontics, preventive, and restorative dentistry to transform resins into hard, highly cross-linked materials. 1 2 3 However, few dental schools teach much about the complex process of resin photopolymerization, resin chemistry, or the curing light. Consequently, most dentists do not know the importance of knowing if the LCUs they are using can deliver an acceptable light output, 4 5 6 7 8 and many do not know how to correctly light cure the resin. 1 4 5 6 7 9 10 11 12

Most dental LCUs emit electromagnetic radiation at wavelengths between 400 and 500 nm (mostly blue light). 2 7 13 The photons emitted provide the energy required to activate the photoinitiator systems used in most dental resins, usually a camphorquinone/amine system. 13 14 15 If an insufficient amount of energy is delivered, or if the light is at the wrong wavelength, this can lead to a significantly undercured resin that can contribute to poor bond strengths, poor mechanical properties, secondary caries, pain, pulp necrosis, and ultimate failure of the product. 16 17 18 19 20 In addition, if the resin-based composite (RBC) does not reach a sufficient degree of monomer conversion, it is more likely to leach undesirable substances into the mouth. 17 21 22

Reducing the time spent on each dental appointment allows more patients to be seen, more restorations to be placed, and a greater income for the dentist. To achieve this goal, some manufacturers are promoting that their curing lights can cure the resin in as little as 1 second. 23 24 LCUs using a laser as the light source have been available for several years, but these were large units. 25 26 Recently, a compact battery-operated laser diode LCU has been introduced. The manufacturer claims this unit can photo-cure 2 mm of RBC in a 1-second light exposure. 23 For RBCs increments thicker than 5 mm, they recommended using three exposures, each lasting 1 second. 23

Many studies have evaluated the depth of cure of RBCs, but most only evaluate the RBC at the center of the specimens directly under the center of the light. 26 27 28 Very few studies look at how well the LCU can photo-cure specimens that represent the size of a restoration in a molar tooth. Those that do, report that while the RBC at the center is well cured, the RBC under the outer regions of the LCU tip is less well photo-cured. 29 30 31 This may be due to the mold, the irradiance beam profile from the light, or both effects. 29 30 31 The International Organization for Standardization (ISO) 4049 dentistry polymer-based filling, restorative materials standard 32 uses a simple test to determine the depth of cure at the center of the RBC specimen. A cylindrical metal mold that is 10 mm deep and 4 mm in diameter is filled with RBC. The RBC is only exposed to light from one side, and the RBC is removed from the mold. Then, the uncured or only partially cured material is immediately scraped away with a plastic instrument. The length of the remaining cylinder of hard RBC is measured, and the ISO depth of cure is determined by dividing the measured length by 2. However, the ISO 4049 test has many limitations. 33 34 35 For example, the 4-mm diameter metal mold is metal, it does not provide any insight into the effects of light beam inhomogeneity, and the size of this metal mold does not represent contemporary restorative dentistry. Today's dentists routinely restore molar teeth that are on average 11.0 mm in mesiodistal length and have a 10.5-mm buccolingual width with RBCs. 36 Also, the scraping method specified in ISO 4049 32 may introduce operator bias. An alternative method has been proposed that uses a no-touch solvent dissolution method and eliminates the operator effect. 35 37 38

This study determined the ability of five currently available LCUs to photo-cure conventional RBCs photo-activated in a class II mold that represented a large restoration in a molar tooth. 30 The hypothesis was that all the LCUs would be equally effective in curing all four RBCs tested.

## Methods and Materials

This blinded study and survey was conducted using 108 samples made from 4 different RBCs that had been photo-cured using 5 different LCUs ( Table 1 ).

**Table 1:** Composite materials used in the study

Composite Resin	Manufacturer	Lot	Shade
SimpliShade Universal	Kerr Corporation, Orange, CA, USA	8086055	DK
Transcend	Ultradent Products Inc., South Jordan, UT, USA	RN776	UB
Omnichroma	Tokuyama Dental Corporation, Tokyo, Japan	042E41	Universal
Filtek Universal Restorative	3M, St Paul, MN, USA	NE15442	A2

These RBCs were photo-activated with five different curing lights, four were light-emitting diode (LED), and one was a laser LCU( Table 2 ) using various exposure times. The study used these LCUs as recommended by the various companies and what might be popular with clinicians after reading advertisements ( Table 2 ). The power values obtained from each LCU were measured using a laboratory-grade spectroradiometer attached to an integrating sphere.

**Table 2:** Light-curing units and settings used in the study

Light-curing unit	Serial number	Manufacturer	Type	Wavelength (nm)	Mode tested	Claimed irradiance (mW/cm ^2^ )
DeepCure	939112012777	3M, St. Paul, MN, USA	Single peak wavelength LED	430–480	1 × 10 s (Standard)	1,470 (–10% / +20%)
					2 × 10 s (Standard)	
PinkWave	00380H	Vista Dental Products, Racine, WI, USA	Multiple peak wavelength LED	395–900	2 × 10 s (Standard)	> 1,515
					1 × 3 (Boost)	> 1720
PowerCure	1428005297	Ivoclar Vivadent, Schaan, Liechtenstein	Multiple peak wavelength LED	385–515	2 × 10 s (high)	1,200
					1 × 3 s (3 s Cure)	3,000
VALO Grand	T10172	Ultradent, South Jordan, UT, USA	Multiple peak wavelength LED	395–480	2 × 10 s (Standard)	1,000
					2 × 3 s (Xtra Power)	3,200
Monet	00249	AMD Lasers, West Jordan, UT, USA	Single peak wavelength laser	450 ± 5	1 × 1 s	2,000–2,400

Abbreviation: LED, light-emitting diode.

Using previously described methods, 3 39 the tip of each LCU was placed at the 16-mm diameter entrance into the 6” integrating sphere (Labsphere Inc., North Sutton, New Hampshire, United States) that was coupled to a fiber optic Flame spectrometer (Ocean Insight, Orlando, Florida, United States). This 16-mm diameter aperture was large enough to capture all the light from the LCUs. An internal traceable light source, SCL 600 (Labsphere Inc.), calibrated the system before beginning the measurements. The emission spectra were also recorded from the LCUs. The beam profile of each LCU tip was recorded using a laser beam profiler (USB-L070, Ophir-Spiricon, Logan, Utah, United States) using previously described methods. 31 The BeamGage software (Ophir-Spiricon) produced color-coded profile images where red represents high (100% or maximum irradiance), and purple represents a low irradiance at the emitting tip of the light guide.

**Fig. 1:**
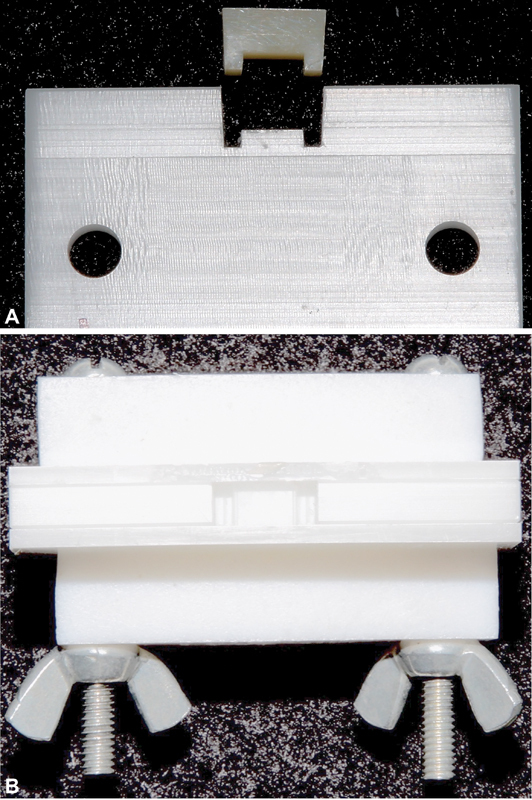
( **A** ) Side view of the mesial-occlusal-distal (MOD) mold and cured composite sample used for calibration. The “legs” represent the proximal boxes. ( **B** ) View of the empty MOD mold from the “occlusal” view.

The various RBC materials were photo-activated in a white plastic Delrin mold that simulated both the optical properties and the size of a 12-mm mesiodistal long and 5-mm deep class II mesial-occlusal-distal (MOD) restoration in a molar tooth ( Fig. 1A and B ). The LCU was clamped over the center of the mold, and the sample was then exposed to light with the light tip 0 mm away from the top surface of the RBC. Three specimens were made in a random order for each combination of RBC and LCU, for a total of 108 cured specimens of RBC.

After photo-curing, the RBC was immediately removed from the mold and immersed in a strong organic solvent (2-Butanone, Sigma-Aldrich, Oakville, Ontario, Canada) at room temperature (20°C) to remove the uncured RBC. After 1 hour, the uncured RBC had been dissolved away. The coded specimens were removed from the solvent and allowed to air dry. They were then placed in a methylene blue stain for 1 hour. After this, they were washed, air dried, and photographed under standard lighting conditions. The partially cured RBC absorbed the methylene blue stain, and the bluer the sample, the less well cured the material appeared. The presence or absence of the mesial and distal “legs” of the RBC down into the proximal boxes was also considered. The lack of or deformed “legs,” in the proximal box area indicated that the RBC was not cured in those areas because the uncured RBC had been dissolved away.

The light beam profiles at the tip of each LCU were measured using a laser beam profiler (Ophir-Spiricon) with a 50-mm focal length lens (SP620U; Ophir-Spiricon). Two blue filters (HOYA UV-VIS colored glass bandpass filter, Edmund Optics, Barrington, New Jersey, United States) and one neutral density filter (Edmund Optics) were required to flatten the spectral response of the charge-coupled device camera. The LCUs were mounted in a fixed orientation and positioned 0 mm distance from the imaging screen, facing toward the camera simulating all the experimental conditions. The images were collected using the beam analyzer software (BeamGage Professional version 6.14; Ophir-Spiricon) and the beam profile images were scaled using the internal tip diameter (mm) of each LCU.

To assess the deidentified images, a Research Electronic Data Capture (REDCap) survey was created. The study data was collected and managed using the REDCap electronic data capture tools hosted at the Medical University of South Carolina (MUSC). 40 41 REDCap is a secure, Web-based software platform that supports data capture for research studies. 40 41 REDCap provides: (1) an intuitive interface for validated data capture, (2) audit trails for tracking data manipulation and export procedures, (3) automated export procedures for seamless data downloads to standard statistical packages, and (4) procedures for data integration and interoperability with an external source.

**Fig. 2:**
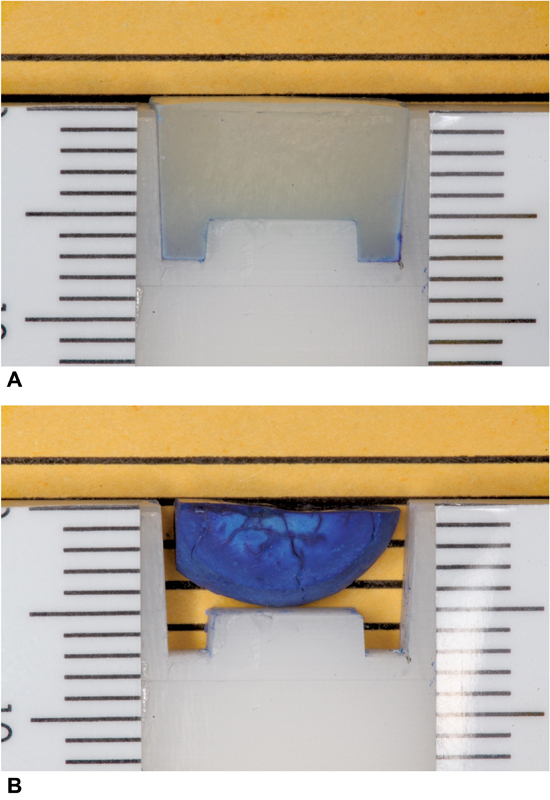
( **A** ) Calibration image – well cured composite. ( **B** ) Calibration image – poor cured composite.

**Fig. 3:**
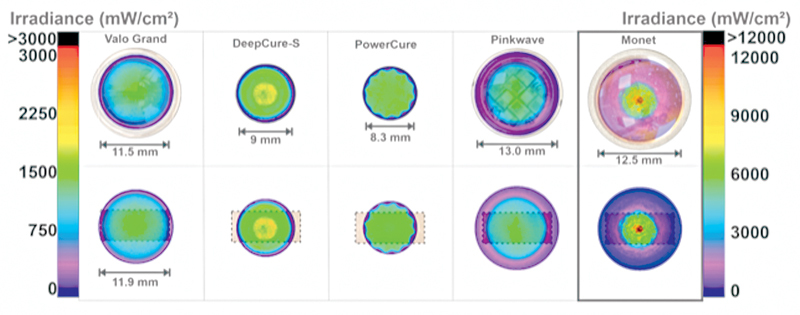
Top image shows the irradiance beam profiles of the curing lights used in the study. Note: The irradiance scale is different for the Monet laser (up to 12,000 mW/cm ^2^ ). Light tip diameter = internal optical diameter from where light is emitted. Lower images show the same beam profile superimposed over the 12-mm long mesial-occlusal-distal (MOD) mold (curing area).

The REDCap survey was sent out to participants, who then blindly reviewed the deidentified sample images and answered a question about how well they thought the RBC in each image had been photo-cured based on the presence, change, or absence of the “legs” of the sample and how much blue was present in the sample images as compared to the blinded image showing a well-cured sample. An image of a well-cured sample ( Fig. 2A ) and a poor cure ( Fig. 2B ) was used to calibrate all who participated prior to them beginning the survey. They were instructed to use this image as the example of a well-cured sample as they evaluated the samples presented to them in the survey. The participants were asked to rank the quality of the RBC cure for each sample from 1 to 5 numerical values, with 1 representing what the observer thought was a poor cure (very blue, legs radically changed) as in Figs 2B and 3 , and 5 representing a good cure (very little to no blue legs intact) as in Fig. 2A .

The survey was sent out to 306 MUSC-James B. Edwards College of Dental Medicine faculty, residents, D4, and D3 students. The results of this blinded survey were then tabulated and analyzed to determine the respondent's impression of the cured MOD samples and for a subjective assessment of the ability of the various LCUs to photo-cure the RBCs.

Quest Graph ANOVA Calculator (AAT Bioquest, Inc., August 2, 2022; https://www.aatbio.com/tools/anova-analysis-of-variance-one-two-way-calculator ) was used to run a one-way analysis of variance (ANOVA) test for comparison among groups. The data distribution was analyzed for normality testing with the Shapiro–Wilk test. Further stratification was performed using the pairwise *t* -test (pooled standard deviation [SD], Benjamini & Hochberg (BH) adjusted). Finally, Welch's one-way ANOVA and Kruskal–Wallis rank tests were run to determine significance. For these tests, *p* -values of ≤ 0.05 were considered significant.

## Results

**Fig. 4:**
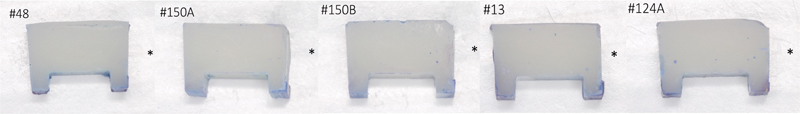
The top five best cured samples (in descending order). (Please place Sample image in order Samples 1-5).

**Fig. 5:**
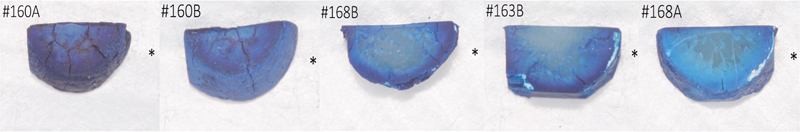
The five least well cured samples (in ascending order). (Please place Sample images in order Samples 10-6).

A total of 63 (20.5%) completed surveys were received. The 108 images of the cured RBCs were then compared by tabulating the average of the survey response to curing effectiveness. The averages were then ranked from the best to the least well-cured sample responses. The 10 best-cured sample images are in descending order, and the curing times, LCU used, and materials are reported in Table 3 and the five best illustrated in Fig. 4 , respectively. The 10 least well-cured RBCs in descending order and the exposure times, lights used, and materials are reported in Table 4 , and the five least well cured are illustrated in Fig. 5 , respectively.

**Table 3:** Ten best cured samples lights and materials were found to be at 2 × 10 seconds

10 Best	Sample	Light	Material	Mean values of all respondents
1	48	PinkWave	Omnichroma	4.5238
2	150A	PinkWave	Omnichroma	4.4762
3	150B	PinkWave	Omnichroma	4.4127
4	13	DeepCure	Omnichroma	4.2698
5	124A	VALO Grand	Omnichroma	4.2698
6	66	VALO Grand	Omnichroma	4.2063
7	171A	PowerCure	Omnichroma	4.0635
8	171B	PowerCure	Omnichroma	4.0000
9	68	VALO Grand	Transcend	3.9683
10	16	DeepCure	Filtek	3.9365

Note: Mean values on a scale of 1 to 5 with 1 being a poor cure and 5 being well cured.

**Table 4:** Bottom ten cured samples and lights (descending order)

10 Worst	Sample	Curing time (s)	Light	Material	Mean values of all respondents
10	144B	1 × 3	PinkWave	SimpliShade	1.1905
9	82	1 × 1	Monet	Omnichroma	1.1613
8	163A	1 × 1	Monet	Filtek	1.1587
7	144A	1 × 3	PinkWave	SimpliShade	1.1429
6	83	1 × 1	Monet	SimpliShade	1.127
5	168A	1 × 1	Monet	SimpliShade	1.1111
4	163B	1 × 1	Monet	Filtek	1.1111
3	168B	1 × 1	Monet	SimpliShade	1.0952
2	160B	1 × 1	Monet	Transcend	1.0476
1	160A	1 × 1	Monet	Transcend	1.0317

Note: Mean values on a scale of 1 to 5 with 1 being a poor cure and 5 being well cured.

The survey found that the top three scores were obtained when the PinkWave (Vista Dental Products) was used to photo-cure Omnichroma (Tokuyama Dental Corporation). Of note, Omnichroma ranked in the top 8 of the top 10 ranked cures in this study, working very well with the 3M Elipar DeepCure-S (3M), VALO Grand Cordless (Ultradent Products, Inc), and Bluephase PowerCure (Ivoclar) lights. In addition, all lights in the top 10 ranked combinations had received 2 to 10-second light exposures.

The bottom 10 cured samples had been exposed to light for either 1 second (Monet) or 3 seconds (PinkWave). The Monet (AMD Laser), with a 1-second cure, was ranked the worst in 8 of the 10 specimens of cured RBCs. Overall, it also ranked poorly with every RBC used in this study. The PinkWave also used for 3 seconds also ranked poorly, particularly with SimpliShade (Ivoclar Vivadent).

One-way ANOVA summary ( Table 5 ) comparing the four LEDs and laser curing devices at the nine settings is listed in Table 2 . A highly significant *p* -value of 1.3246e-24 was obtained since a value of “e” is to a power of 10.

**Table 5:** One-way ANOVA calculation

Group	Degrees of freedom (DF)	Sum of squares (SS)	Mean square (MS)	*F* -statistic	*p* -Value
Between groups	8	71.072	8.884	32.7812	1.3246e-24
Within groups	99	26.8299	0.271		
Total	107	97.9019			

Abbreviation: AVOVA, analysis of variance.

Note: *p* -Value 1.3246e-24 = 1.3246 × 10 ^−24^ < 0.05 indicating a significant difference. : ANOVA summary.

**Fig. 6:**
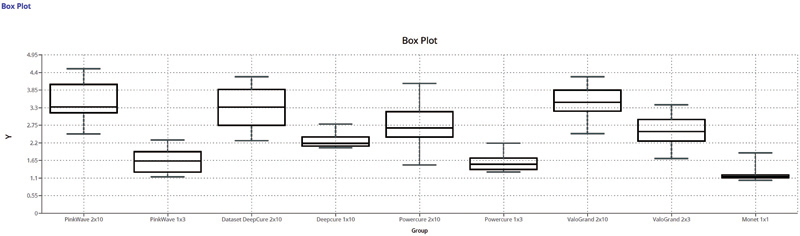
Box plot showing the results from all the light-curing units and modes of cure. The data depicts the five-number summary as the minimum, first quartile, median, third quartile, and maximum values.

Shapiro–Wilk normality test results ( Table 6 ) confirmed that the data was normal. This is further illustrated by applying a normal Q-Q plot ( Fig. 3 ). Pairwise *t* -test (pooled SD, BH adjusted) comparison results ( Table 7 ) provided significant *p* -value results of < 0.05 for 28 of the 36 comparisons tested. Welch's one-way ANOVA results ( Table 8 ) and Kruskal–Wallis rank test ( Table 9 ) also showed a significant difference in the findings. These differences are further illustrated in the box plot ( Fig. 6 ).

**Table 6:** Shapiro–Wilk normality test results

Parameter	Value
*W*	0.9774
*p* -Value	0.0621

Note: *p* -Value > 0.05 not significant. Data is normal.

**Table 7:** Pairwise *t* -test (pooled SD, BH adjusted) comparison results

	PinkWave 2 × 10	PinkWave 1 × 3	DeepCure 2 × 10	DeepCure 1 × 10	PowerCure 2 × 10	PowerCure 1 × 3	VALO Grand 2 × 10	VALO Grand 2 × 3
PinkWave 1 × 3	2.4661e-13	−	−	−	−	−	−	−
DeepCure 2 × 10	0.2391	8.1556e-11	−	−	−	−	−	−
DeepCure 1 × 10	2.5232e-7	0.0043	0	−	−	−	−	−
PowerCure 2 × 10	0.001	0.0000018561	0.0325	0.0346	−	−	−	−
PowerCure 1 × 3	9.7362e-14	0.8129	2.4893e-11	0.002	6.3062e-7	−	−	−
VALO Grand 2 × 10	0.9221	3.4497e-13	0.2729	3.5949e-7	0.0013	1.2728e-13	−	−
VALO Grand 2 × 3	0	0.0001	0.0027	0.2155	0.3845	0	0.0001	−
Monet 1 × 1	5.2168e-17	0.0594	1.1538e-14	0.0000048365	2.8868e-10	0.1026	5.2168e-17	1.9787e-8

Abbreviation: SD, standard deviation.

Note: Highlighted values are *p* -value < 0.05.

**Table 8:** Welch's one-way ANOVA results

Parameter	Value
*F*	44.4721
DF (numerator)	8
DF (denominator)	40.8844
*p* -Value	9.243e-18

Abbreviations: AVOVA, analysis of variance; DF, degrees of freedom.

Note: *p* -Value < 0.05 indicating significant difference.

**Table 9:** Kruskal–Wallis rank test

Parameter	Value
Kruskal–Wallis chi-squared	81.5013
DF	8
*p* -Value	2.4368e-14

Abbreviation: DF, degrees of freedom.

Note: *p* -Value < 0.05 indicating significant difference.

## Discussion

Light-cured RBCs and LCUs have become an essential part of modern dentistry. The importance of using the correct technique and the right LCU cannot be overlooked. Yet, the dental professional often may not consider the consequences of using an exposure time or a curing light that cannot cure the bottom of their RBC restorations because the top feels hard. The requirement to phase down the use of dental amalgam due to the Minamata agreement 42 43 has driven the dental industry to create improved resin-based restorative materials. Since there has always been a desire to cure RBCs faster, some manufacturers claim that their LCU can cure RBCs in just 1 second. Alternatively, using bulk-cured RBC materials can be successful 44 45 46 and can reduce the time required to place direct resin restorations. 47 Such promises make the fast curing RBC or LCU very attractive to the purchaser who wants to save time and generate more income.

However, this desire to photo-cure RBCs faster and faster can have a detrimental effect on the outcomes of the restorative process. Moving from conventional RBCs that should be photo-activated in increments that are at most 2 mm thick, bulk-cured materials can be photo-activated in increments that are 4 to 6 mm thick, depending on the brand of RBC. While this time saving is advantageous for the dental clinician, its potential unintended consequences could be dire. 22 48 49

Three RBCs used in this study were popular products, and one RBC has yet to be released. Omnichroma (Tokuyama Dental Corporation) is reported to be “the first universal composite to shade match any tooth color… (which is) strong, durable, and versatile… (to) streamline the restorative process.” Filtek Universal (3M) is reported to be “designed to make single-shade restorations easier…with just 8 designer shades, and an XW shade…(to) cover all 19 Vita classical shades and bleach shades.” SimpliShade (Kerr Corporation) is reported to be a “Simplified Universal composite with Adaptive Response Technology…featuring three shades (light, medium and dark) … (to make) quick and easy to match all 16 Vita Classical shades.” Transcend is a new RBC that will soon be released from Ultradent Products, Inc. According to the company, it contains four different resin monomers in various percentages described as functional methacrylates. It has a filler weight percentage of approximately 77.5%, containing a primary photoinitiator (camphorquinone) and another proprietary photoinitiator.

This study was not designed to measure the hardness or degree of conversion of the RBCs. Instead, it was intended to provide visual images to illustrate what was cured and resistant to solvent removal and what was uncured material. The white Delrin MOD mold was approximately as large as a clinical MOD restoration. The RBC samples produced provide observational insights regarding how well these RBC materials are photo-activated in the controlled environment. The result was a highly visual, apples-to-apples comparison that can be easily translated to clinical practice. The ISO 4049 test determines the depth of cure by photo-curing 4-mm diameter specimens, immediately measuring the length of the remaining hard RBC, and then dividing that number by 2. Since all the RBCs used in this study should provide at least a 2-mm depth of cure, the MOD mold must be at least 4 mm deep. In fact, the mold used was 5 mm deep at the proximal boxes, and it showed that at least some combinations of RBCs and LCUs could produce a MOD restoration that was intact and resistant to a strong organic solvent. The results from this blinded survey help emphasize that different LCUs and RBCs behave differently. As expected, all lights tested photo-cured the top of the RBC directly under the center of the light tip, but at the extremes of the preparation, the RBC specimens were not so well cured. This outcome may be due to the size of the LCU tip, and the amount of light energy produced. Considering the difficulty making well-cured composite restorations under the controlled environment in the laboratory, where there are no concerns for effective isolation or the LCU potentially “drifting” away from the RBC or any other clinical issues, how can dentists create these critically important restorations *in vivo* ?

This novel study is the first to visually examine the 1-second cure claims using the Monet laser LCU and other LED curing lights in a mold representing a tooth cavity. Although one previous article has reported that a blue diode laser can achieve a greater depth of cure 26 than a multiple wavelength LCU when they emit at the same radiant exposure, the laser was used “off label” for 20 seconds. It produced a temperature of 107.8°C in the 2-mm thick RBC. Thus, the results of that article can be dismissed as not clinically relevant because, hopefully, nobody would use a laser for 20 seconds. A recent article 50 evaluated the depth of cure of 10 contemporary RBCs by three LCUs including the Monet light at 1 second. That study found that all the LCUs tested were able to cure those composites, however, the laser had the shallowest depth of cure. This agrees with the results of this visual survey.

Additionally, data obtained in the pairwise *t* -test ( Table 7 ) showed a significant difference between the Monet used for 1 second compared to 2 × 10 second light exposures from the PinkWave, DeepCure, PowerCure, and VALO Grand and even when compared to the 1 × 10 second cure of the DeepCure. This illustrates the potential dangers of not delivering sufficient energy to cure the RBCs even when using the same light and on the same materials. Therefore, using the lights evaluated in this study, we cannot afford to take shortcuts in our process of curing RBC materials. The top surface the dentist can touch will typically appear hard; however, this study demonstrates that the bottom may not be hard. A dentist may presume that when the LCU “lights up,” all of the RBC will be photo-cured. In reality, only the RBC directly under the light tip receives sufficient irradiance and energy to photo-cure in the exposure time used.

**Fig. 7:**
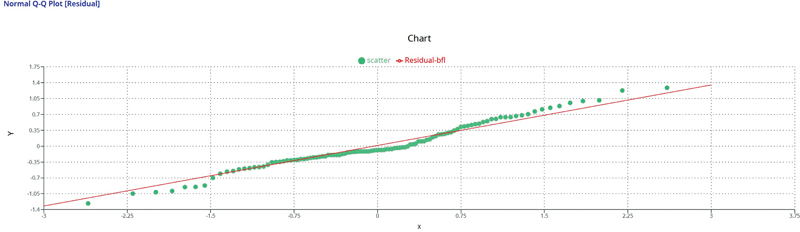
Normal Q-Q plot (residual). The data meets homoscedasticity and normal assumptions of linear regression.

This blinded survey removed any possibility of bias since the samples were blinded in their creation at one university, and the survey results were obtained at another university. The 63 participants also were blinded to this information. They provided their opinions based solely on the description of the process used to create the samples and answered the questions based on these descriptions (how the shape of each sample differed from the ideal sample and how blue the samples were). In addition, the RBC specimens were viewed in random order. Additionally, the data of this study, as analyzed by the Shapiro–Wilk test ( Table 6 ), was normalized as well as the normal Q-Q plot ( Fig. 7 ).

Evaluating the LED/laser light comparisons, in the pairwise *t* -test ( Table 7 ) and the Beam profiles and areas that could be photo-cured with the size of the light tips used ( Fig. 5 ) shows a significant difference between three of the LED lights used for 20 seconds and the other LCUs settings used for shorter exposure times. This can be attributed to the greater amount of energy delivered (more photons) over a larger area by those LED units in the RBCs curing process. On the other hand, the Monet 1 × 1 was not significantly different from the PinkWave 1 × 3 second and PowerCure 1 × 3 second, most likely because of the lower amount of energy delivered to the samples using these exposure conditions.

The beam profiles of the various LCUs ( Fig. 3 ) show that the four LED units have a wide beam profile covering most of the restoration with useful light. However, the PowerCure 2 × 10, with its 8.3-mm internal diameter, delivered less energy; hence, the specimens made with this light were not as well cured as the other three LED lights used for 20 seconds in the pairwise *t* -test. The curing tip diameter is also important when curing the samples 12 mm wide and 5 mm deep in this study. This study's findings are especially important when curing more extensive restorations intraorally. Multiple light exposures are required to fully cover the RBC with light from the LCU.

Also, as illustrated in Fig. 3 , even though it has a 12.5-mm light tip, the laser curing light has a much smaller tip beam profile than the other LCUs. When the images of the MOD restorations are combined with the beam profiles, it appears that the curing area in this beam profile is less than half the diameter of its light tip. This is supported by the results of this study. The Monet only cured the RBCs directly under the center of the light tip, and it failed to cure the RBCs in the proximal boxes. Also, note the hot spots of high irradiance at the center of the Monet light tip illustrated by the red colors in the beam profiles. This area potentially could create an overheating of the composite materials. The lower irradiance (purple and pink) seen in the outer tip regions of the laser curing light may contribute to the low curing results seen in this study. All LEDs used for the shorter exposure times yielded significant differences and worse photo-curing results.

## Conclusion

This novel study is the first to test the 1-second cure claims using the Monet laser LCU for the recommended exposure time and other LED curing lights used for shorter intervals in a mold representing a tooth. When used for 1 second, the laser curing device did not photo-cure conventional RBC materials as did the LED curing lights used for 10 seconds.
